# RNA partitioning into stress granules is based on the summation of multiple interactions

**DOI:** 10.1261/rna.078204.120

**Published:** 2021-02

**Authors:** Tyler Matheny, Briana Van Treeck, Thao Ngoc Huynh, Roy Parker

**Affiliations:** 1 Department of Biochemistry, University of Colorado, Boulder, Colorado 80309, USA; 2 Howard Hughes Medical Institute, Chevy Chase, Maryland 20815, USA

**Keywords:** stress granules, RNA partitioning, RNA-binding proteins, RNP condensate, G3BP1

## Abstract

Stress granules (SGs) are stress-induced RNA–protein assemblies formed from a complex transcriptome of untranslating ribonucleoproteins (RNPs). Although RNAs can be either enriched or depleted from SGs, the rules that dictate RNA partitioning into SGs are unknown. We demonstrate that the SG-enriched NORAD RNA is sufficient to enrich a reporter RNA within SGs through the combined effects of multiple elements. Moreover, artificial tethering of G3BP1, TIA1, or FMRP can target mRNAs into SGs in a dose-dependent manner with numerous interactions required for efficient SG partitioning, which suggests individual protein interactions have small effects on the SG partitioning of mRNPs. This is supported by the observation that the SG transcriptome is largely unchanged in cell lines lacking the abundant SG RNA-binding proteins G3BP1 and G3BP2. We suggest the targeting of RNPs into SGs is due to a summation of potential RNA–protein, protein–protein, and RNA–RNA interactions with no single interaction dominating RNP recruitment into SGs.

## INTRODUCTION

Stress granules (SGs) are ribonucleoprotein (RNP) assemblies that form during stress when translation initiation is limited (Panas et al. 2016; Protter and Parker 2016). SGs are of interest because they play roles in the stress response, are related to similar RNP granules in neurons and embryos, share components with toxic aggregates observed in degenerative disease, and affect viral infections as well as cancer progression (Kedersha et al. 2013; Kim et al. 2013; Li et al. 2013; Reineke and Lloyd 2013; Anderson et al. 2015; Somasekharan et al. 2015).

Based on super-resolution microscopy, mammalian SGs formed during arsenite stress are nonuniform in nature and contain local regions of protein and RNA concentration, referred to as SG cores (Jain et al. 2016; Wheeler et al. 2016; Niewidok et al. 2018). In addition to this imaging criteria, cores are defined by their biochemical stability in cell lysates (Jain et al. 2016; Wheeler et al. 2016). This has allowed for SG core purification from U-2 OS cells expressing GFP-G3BP through differential centrifugation and GFP pulldown (Wheeler et al. 2017; Khong et al. 2018). Purification of SG cores led to the discovery of a diverse SG proteome composed of numerous RNA-binding proteins (RBPs) forming a dense protein–protein interaction network (Jain et al. 2016). Similar SG protein composition was also detected by proximity labeling methods (Markmiller et al. 2018; Youn et al. 2018). The fact that SGs include a protein–protein interaction network is consistent with the recruitment of proteins to SGs through protein–protein interactions, which might recruit specific mRNAs bound to those RBPs.

An additional possibility is that RNA–RNA interactions contribute to defining the RNA composition of SGs. Consistent with this model, the transcriptome of protein-free in vitro RNA assemblies, formed from total yeast RNA, shows remarkable overlap with the transcriptome of yeast SGs (Van Treeck et al. 2018). Moreover, the abundant RNA helicase eIF4A functions to limit RNA condensation and SG formation by binding RNA (Tauber et al. 2020). This suggests a model wherein RNPs are partitioned into SGs by both RNA–RNA and protein–protein interactions (Van Treeck and Parker 2018).

A striking feature of the SG transcriptome is that there are dramatic differences in the partitioning of RNPs into SGs. For example, RNA sequencing of immunopurified SGs revealed that some RNAs, such as AHNAK or NORAD, are strongly enriched in SGs, while other mRNAs, such as *GAPDH*, are largely depleted from SGs (Khong et al. 2017). Differential partitioning of RNPs into SGs and/or P-bodies has also been seen when RNP granules are fractioned based on particle sorting or differential centrifugation (Hubstenberger et al. 2017; Namkoong et al. 2018; Matheny et al. 2019). These analyses revealed that long, poorly translated transcripts preferentially enrich in SGs (Khong et al. 2017), that AU-rich elements correlated with SG granule fractionation (Namkoong et al. 2018), and that decreased translational efficiency correlated with increased SG and P-body enrichment (Hubstenberger et al. 2017; Khong et al. 2017; Matheny et al. 2019). Taken together, these observations suggest that partitioning of RNPs into SGs could be promoted by protein- or RNA-based interactions, affected by overall length of the RNA, and inhibited by the association with ribosomes. However, the relative importance of these interactions, their required valency, and how they might contribute to the RNA composition of, and organization within, SGs is unknown.

Herein, we examine the rules that affect the partitioning of RNAs into SGs. We first show enrichment into SGs is a dominant property as a chimera between the highly enriched NORAD RNA and a SG excluded reporter RNA is enriched in SGs. Deletion analysis revealed the enrichment of NORAD in SGs is due to multiple sequence elements that act in an additive manner. In addition, we examined how proteins influence the recruitment of RNAs to SGs by determining how tethering of RBPs found in SGs to a reporter transcript affects mRNA enrichment in SGs and how deletion of the major SG RBPs G3BP1 and G3BP2 affects RNA recruitment to SGs. We show that artificially tethering multiple G3BP1, FMR1, or TIA1 proteins to a luciferase reporter transcript increases the SG localization of a luciferase RNA reporter in a dose-dependent manner. However, based on curve fitting of these tethering experiments, we predicted that removal of G3BP proteins from the cell would only have a limited effect on the RNA composition of SGs. Testing this hypothesis, we demonstrate that the RNA composition of sorbitol-induced SGs is very similar in wild type (WT) and ΔΔG3BP1/2 cell lines. Taken together, our results indicate that while G3BP1 binding can promote RNA targeting to SGs, it is not required for SG localization. We suggest a model in which RNA localization to SGs arises through the summation of many RNA–RBP and RNA–RNA interactions, which each individually play a small role in localization, but through synergistic effects lead to the observed RNP partitioning into RNP granules.

## RESULTS

### Luciferase mRNA as a reporter for SG partitioning in mammalian cells

The firefly luciferase was used as an RNA reporter to assess how different elements affect RNP partitioning into SGs for two reasons. First, luciferase is not endogenous in mammalian cells, thus, we can eliminate the endogenous background in our analysis. Second, single molecule fluorescent in situ hybridization (smFISH) analyses revealed that luciferase mRNA is poorly enriched in SGs, with ∼15% of its cytoplasmic RNA molecules in SGs upon an hour of sodium arsenite treatment (Supplemental Fig. S1). Using this reporter system, with a downstream SV40 poly(A) signal, we examined how adding sequences or binding sites for RBPs affected SG partitioning.

### NORAD RNA contains dominant elements that dictate SG partitioning

**FIGURE 1.**
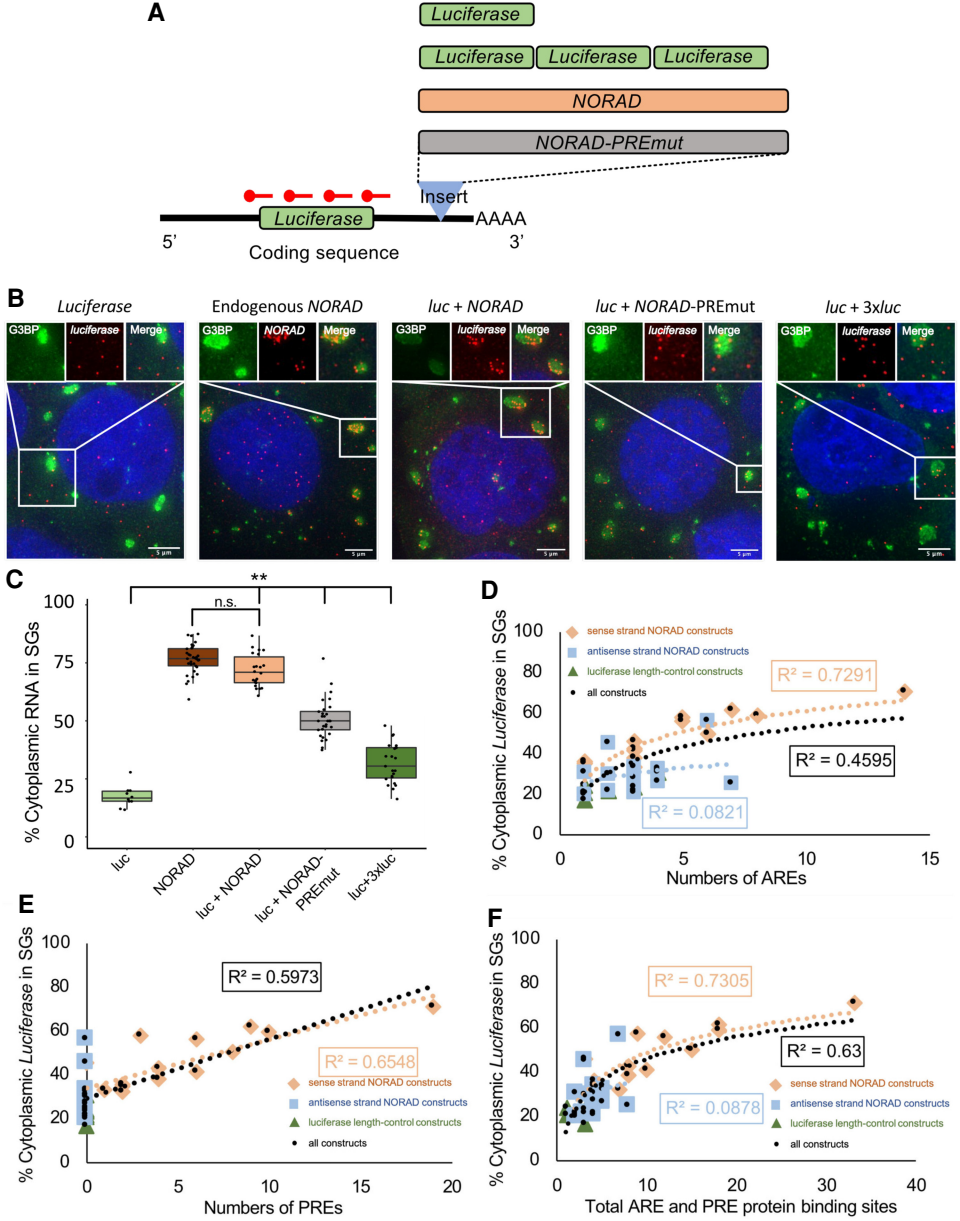
NORAD increases luciferase RNA enrichment within SGs. (*A*) Cartoon depicting chimeric luciferase reporter constructs with different inserts in the 3′-UTR. PREmut is the NORAD sequence with 18 of the PREs mutated. The 3× luciferase was inserted as an approximate length control for NORAD. (*B*) smFISH of luciferase RNA for different constructs during arsenite stress. The endogenous NORAD RNA was imaged with smFISH probes to NORAD, all other constructs were imaged with smFISH probes to luciferase. Scale bar = 5 µm. (*C*) Boxplot of the enrichment of endogenous NORAD and luciferase with different 3′-UTR inserts in SGs. (**) *P* < 1 × 10^−4^. Each dot represents a single cell. (*D*) Correlation between SG enrichment and number of AREs. (*E*) Correlation between SG enrichment and number of PREs. (*F*) Correlation between SG enrichment and total number of AREs and PREs. For (*D*–*F*): Orange diamonds are sense-NORAD constructs, light blue squares are antisense-NORAD constructs, dark green triangles are luciferase length control constructs, and black circles are all the constructs.

While it is assumed that partitioning of RNPs into SGs is a dominant trait, to our knowledge this has never been tested. To determine if SG partitioning is a dominant trait, we inserted the SG-enriched NORAD RNA into the 3′-UTR of the luciferase reporter mRNA. As assessed by smFISH, we observed that ∼71% of the chimeric luciferase-NORAD RNAs were recruited to SGs, similar to the endogenous NORAD RNA (76.7%) and significantly increased compared to the luciferase reporter (16.7%) (Fig. 1A–C). Thus, SG recruitment of RNPs is a dominant property of nontranslating, cytoplasmic RNAs.

To determine whether one or more elements in the NORAD RNA were promoting SG partitioning, we constructed luciferase mRNAs with either each half, each of the four quarters, or each eighth of the NORAD RNA inserted into the same site in the 3′-UTR.

We observed that essentially any piece of NORAD could increase the recruitment of the luciferase reporter into SGs with the average increase correlating with the size of the insert (Supplemental Table S1). For example, either half of NORAD yielded ∼60% reporter enrichment within SGs, each quarter gave between ∼55% and 35% recruitment, and each eighth gave between 22% and 50% recruitment (Supplemental Table S1). These increases are not solely due to increased length since insertion of additional luciferase sequences of similar lengths in the same position within the 3′-UTR gave only limited increases in SG recruitment (Supplemental Table S1). Indeed, the relationship between length and SG enrichment only gave a small positive slope, suggesting that length, per se, is not the major driving force in SG enrichment and that the previously defined length dependence is likely due to long transcripts harboring more RBP sites and/or RNA–RNA interaction motifs. Similarly, insertion of antisense sequences from NORAD typically resulted in less recruitment of the reporter into SGs than corresponding sense-strand inserts (Supplemental Table S1). The limited correlation with length is consistent with the SG transcriptome in which there is an overall length determinant; however, mRNAs of similar length can show differences in their partitioning into SGs (Khong et al. 2017). This suggests that NORAD contains multiple sequence-specific elements, independent of overall length, that can increase partitioning into SGs. These elements may increase SG localization through interaction with multivalent RBPs, or through increased propensity of these RNA sequences to undergo RNA-mediated assembly.

We compared the features of the NORAD-luciferase chimeric RNAs to determine if any feature strongly correlated with SG enrichment. We observed only a limited correlation between SG recruitment and length or GC content (Supplemental Fig. S2). In the NORAD RNA there are 13 AU-rich elements (AREs) with an AUUUA motif and 19 Pumilio recognition elements (PREs), which can bind Pumilio and other RBPs (Lee et al. 2016; Tichon et al. 2016). We observed some correlation of SG enrichment in each construct with either the numbers of AREs, PREs, or their summation (Fig. 1D–F). There are likely multiple RBP binding sites in these segments that lead to differential localization. The presence of correlation implied that ARE- and PRE-binding proteins may contribute to the SG enrichment of NORAD.

To test whether the 19 PREs are important for NORAD recruitment in SGs, we generated a chimeric mRNA with the NORAD sequence with 18 PREs mutated to limit Pumilio (and possibly other proteins) binding, into the luciferase 3′-UTR. We observed a significant decrease in luciferase reporter, from 71% to 50%, in SGs (Fig. 1B,C). Since cells lacking Pumilio proteins can still robustly accumulate NORAD in SGs (Namkoong et al. 2018), this suggests that the PREs can promote SG enrichment either as an RNA element, or potentially by binding other RBPs in addition to Pumilio, such as SAM68, which has binding sites in or near many of the PREs (Tichon et al. 2018).

Taken together, these observations argue that NORAD contains multiple elements that can increase an mRNAs partitioning into SGs. Moreover, the correlation of enrichment with protein binding sites suggests that at least some of the SG enrichment will be due to RBPs targeting mRNAs to SGs.

### Tethering G3BP1 to a reporter mRNA increases the reporter RNA's enrichment within stress granules

To directly examine how the interactions with mRNA-binding proteins affect mRNA partitioning into SGs, we next focused on G3BP1 for three reasons. First, G3BP1 is one of the most abundant cytoplasmic RBPs (Itzhak et al. 2016). Second, G3BP1 partitions strongly into SGs (Wheeler et al. 2017). Third, G3BP1, and its paralog G3BP2, are required for SG formation under certain stresses (Tourrière et al. 2003; Kedersha et al. 2016). We hypothesized that if protein–RNA interactions of RBPs play a critical role in localizing transcripts to SGs, increasing the number of G3BP1 molecules bound to a specific mRNA by artificial tethering should lead to increased targeting of that mRNA to SGs.

**FIGURE 2.**
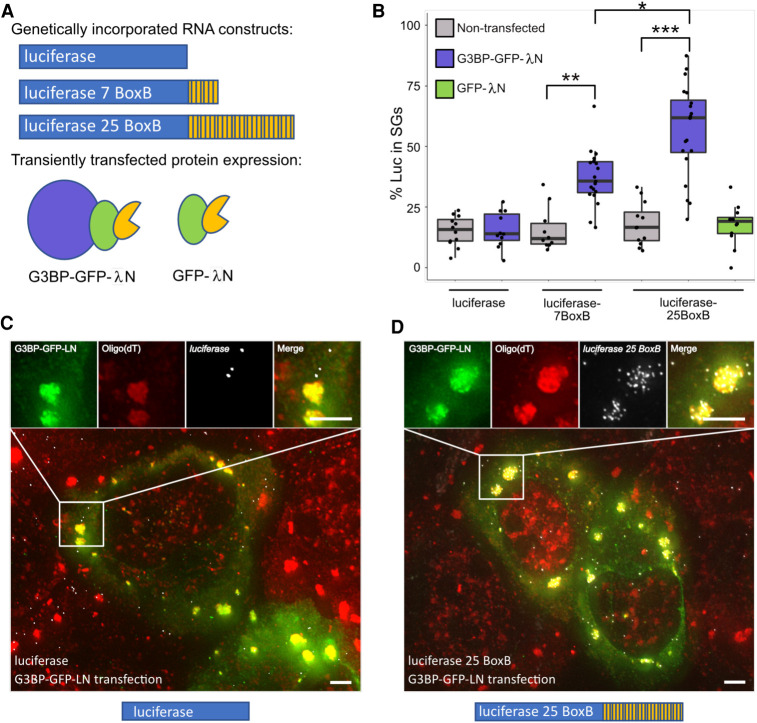
G3BP tethering increases luciferase RNA enrichment within SGs. (*A, top*) Cartoon depicting genomically incorporated luciferase RNA constructs with zero, seven, and 25 BoxB sites in the 3′UTR. (*Bottom*) Cartoon depicting G3BP-GFP-λN and GFP-λN used in tethering experiments. (*B*) Boxplot of the enrichment of luciferase zero BoxB, seven BoxB, and 25 BoxB RNA in nontransfected, G3BP-GFP-λN and GFP-λN transfected cells. (*) *P* < 0.001, (**) *P* < 1 × 10^−4^, (***) *P* < 1 × 10^−7^. Each dot represents a single cell. (*C*) smFISH of luciferase RNA in G3BP-GFP-λN transfected cells during arsenite stress. Scale bars = 2 µm. (*D*) smFISH of luciferase 25 BoxB RNA in G3BP-GFP-λN transfected cells during arsenite stress. Scale bars = 2 µm.

To determine if G3BP1 could increase a given RNA's localization to SGs, smFISH was used to monitor localization of the luciferase RNA reporter with and without G3BP1 tethering sites. We used the λN-BoxB system to artificially tether G3BP1 to the luciferase mRNA (Baron-Benhamou et al. 2004). λN was fused to G3BP1-GFP as well as a GFP control (Fig. 2A). Luciferase mRNA reporters were created with zero, seven, and 25 BoxB sequences in their 3′-UTR and were genomically incorporated into the AAVS locus of U-2 OS cells (Fig. 2A). Reporter RNA localization was assayed in cells transfected with either G3BP1-GFP-λN or GFP-λN and in nontransfected cells.

A key observation was that upon mild to moderate G3BP1-GFP-λN expression, luciferase RNAs with seven or 25 λN binding sites shifted luciferase from a median of 14% partitioning in SGs to ∼36% or ∼62% partitioning, respectively (Fig. 2B–D). Notably, this effect is dose-dependent since mRNAs with 25 BoxB sequences showed increased SG partitioning as compared to mRNAs with seven BoxB sequences (Fig. 2B). Addition of BoxB repeats to luciferase did not affect RNA partitioning to SGs when expressed without any λN protein or when coexpressed with GFP-λN (Fig. 2B). Thus, the observed effect is due to the ability of the transcript to interact with G3BP1 and is not simply due to the increased length of the transcript. Examination of individual transfected cells showed that the reporter partitioning into SGs was similar over a range of G3BP1 concentrations (Supplemental Fig. S3), suggesting the effect is not a result of G3BP1 overexpression. Cells that highly over-expressed G3BP1, which leads to the formation of constitutive SGs (Tourrière et al. 2003), were not included in this analysis. Taken together, these observations demonstrate that, at least during arsenite stress, the presence of multiple G3BP1 proteins on an mRNP can alter the partitioning of that mRNA to SGs in a dose-dependent manner. Therefore, at least some SG RBPs can influence the partitioning of client RNAs to SGs.

**FIGURE 3.**
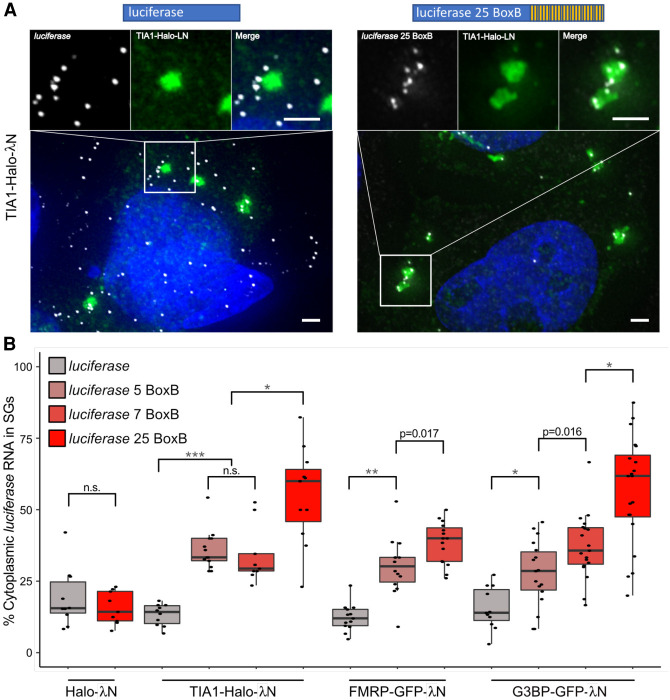
Tethering of TIA1, FMRP, or G3BP increases luciferase RNA enrichment in SGs. (*A*) smFISH of luciferase and luciferase 25 BoxB RNA coexpressed with TIA1-Halo-λN. Scale bars = 2 µm. (*B*) Boxplots depicting enrichment of luciferase zero BoxB, five BoxB, seven BoxB, and 25 BoxB RNA when tethered to G3BP, FMRP, and TIA1 through λN-BoxB interactions. Tethering of Halo-λN to luciferase RNA serves as a negative control. (*) *P* < 0.01, (**) *P* < 1 × 10^−4^, (***) *P* < 1 × 10^−7^. Each dot represents a single cell.

To determine if this effect was unique to G3BP1-GFP-λN, we also tethered GFP-λN or Halo-λN tagged versions of the stress granule components TIA1 or FMRP, and a G3BP1-Halo control to luciferase mRNAs with five, seven, or 25 BoxB sites. We observed that TIA1 and FMRP could recruit the reporter mRNA into SGs to the same extent as G3BP1 and with a similar dose dependent effect (Fig. 3). We were unable to evaluate the effects of tethering FMRP to an mRNA with 25 BoxB sites since this led to very low levels of the reporter being expressed. This suggests that some SG-enriched RBPs can actively recruit client RNAs to SGs or, by creating a high local concentration of SG components and RNA, seed SGs.

### Quantitative estimation of the number of SG interactions with mRNAs

Based on our G3BP1 and TIA1 tethering experiments, we performed mathematical modeling of SG recruitment as a function of the number of RNA–RBP interactions. In our modeling, for a first approximation, we assume all BoxB sites are occupied by G3BP-GFP-λN or TIA1-GFP-λN. Thus, the unmodified luciferase reporter mRNA has an unknown number of interactions, *n*, with SGs, and each BoxB-luciferase reporter has a number of SG interactions that is equal to the sum of the unmodified luciferase SG interactions, *n*, and the number of BoxB sites on that transcript.

**FIGURE 4.**
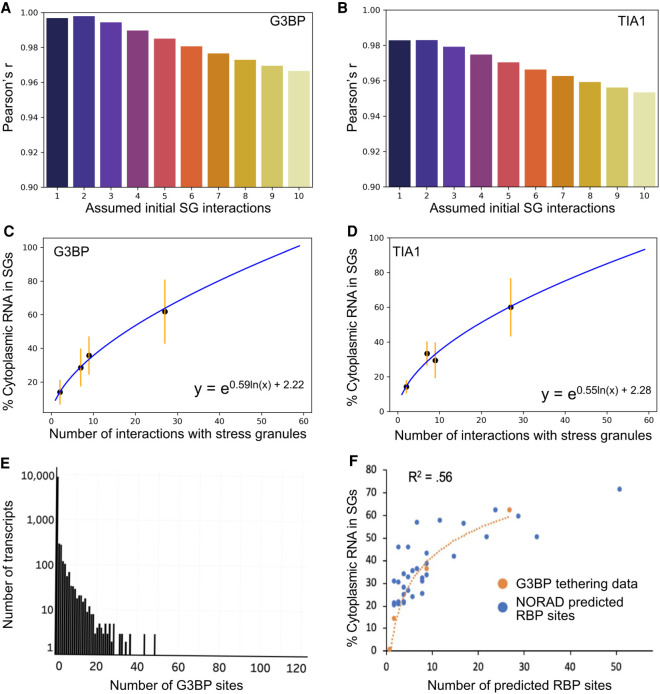
Mathematical modeling of RBP interactions suggest deletion of G3BP may have a limited effect on RNA enrichment in SGs. (*A*) Boxplot depicting Pearson's *r* values for G3BP curve fitting performed using various values of n (initial luciferase-SG interactions prior to the addition of any BoxB sites). (*B*) Same as *A*, but for TIA1 tethering data. (*C*) Curve of best fit for G3BP data. Black dots represent median values of SG enrichment. Orange error bars represent the standard deviation of the data. (*D*) Curve of best fit for TIA1 data. Black dots represent median values of SG enrichment. Orange error bars represent the standard deviation of the data. (*E*) Histogram depicting the number of transcripts that contain different amounts of G3BP eCLIP peaks. Note that the *y*-axis is on a log scale. (*F*) Curve fitting overlaying the predicted summation of PRE, ARE, and SAM68 sites for NORAD fragment transcripts and G3BP tethering experiments.

We performed curve fitting analysis allowing *n* to vary from one to 10 luciferase interactions with SG RBPs prior to the addition of any BoxB sequences. Upon qualitative examination of the data, and given the implicit ceiling of 100% enrichment, we reasoned that a nonlinear fit would be most appropriate (Supplemental Fig. S4A,B). To estimate Pearson's *r* value for each fit, we linearized the data by performing a logarithmic transformation (Supplemental Fig. S4C,D). For both TIA1 and G3BP tethering experiments, curve fitting yielded a fit that showed a maximum R value for the fit in which *n* = 2 (Fig. 4A,B). We observed similar curves for both G3BP1 and TIA1, suggesting that these two proteins have a similar ability to concentrate client RNAs within SGs (Fig. 4C,D).

This analysis highlights a few key principles in RNA recruitment to SGs. First, this result suggests that RNP interactions with a SG will not affect the recruitment of all transcripts to SGs equally. For example, loss of a single SG interaction from an mRNP with 27 total SG interactions would only reduce the expected proportion of mRNAs in SGs from 62% to 61%. In contrast, the loss of one interaction for an mRNP with three SG interactions should reduce its accumulation in SGs from ∼18% to ∼14%. These observations highlight the key point that RNPs with small numbers of interactions with SGs should be affected to a greater extent by changing a single interaction, while RNPs with large numbers of SG interactions should be less affected by loss of a single RNA–RBP interaction.

Second, the maximum effect of a given RBP–RNA interaction on SG partitioning should be relatively small, and much less for RNAs which have many nonredundant RNA–RBP interactions. This second observation is important because eCLIP analysis shows that the majority of transcripts in the cell have 0-3 G3BP binding sites (Fig. 4E; Van Nostrand et al. 2020).

Third, assuming all SG-targeting interactions are similar, one can make an estimate for the number of interactions a given mRNA has with SGs. From this curve, we made a transcriptome-wide first approximation of the number of interactions a given mRNA forms with SGs (Supplemental Table S2). Given the assumptions in this model and the cell to cell variation in SG enrichment, these numbers should not be taken literally, but should be used as “ballpark” estimates.

One assumption in this modeling is that every BoxB site is directly interacting with a G3BP1 or TIA1 molecule. If we assume that only half of the sites are occupied at a given time, then our estimates for the number of interactions would be correspondingly reduced. However, the logic remains valid that the higher the number of interactions an RNA can form with SGs, the less sensitive that RNA will be to perturbations of individual interactions.

### Modeling explains behavior of the NORAD-luciferase constructs

To see if we can understand the NORAD-luciferase chimeric RNA results in terms of different numbers of RBPs, we first estimated the number of proteins bound to each region (Supplemental Table S1). In this analysis, we counted PREs, which serve as binding sites for Pumilio (Lee et al. 2016; Tichon et al. 2016), AREs, which can bind a diverse number of different ARE-binding proteins and have been shown to correlate with SG enrichment (Namkoong et al. 2018), and Sam68 binding sites, which have also been mapped to NORAD (Tichon et al. 2018). We then plotted the number of predicted RBPs bound to each construct versus the SG enrichment. Remarkably, this set of data points fit the curve derived from the tethered G3BP1 RNAs quite well (Fig. 4F). Moreover, this analysis would predict that the PRE mutant, which has 18 deleted RBP binding sites, should change the recruitment of the luciferase reporter from 71% to 61%, near the observed 50%. The deviation between recruitment of the PRE mutant and the predicted value may be due to the PRE mutations disturbing other protein binding sites. Thus, the behavior of both the tethered G3BP1 reporters, and the luciferase-NORAD chimeric mRNAs, can be explained as being due to the summation of multiple interactions that together increase the partitioning of RNPs into SGs.

We also estimated the number of RBPs bound to NORAD segments based on CLIP sites from a database containing a meta-analysis of RBP CLIP studies (Supplemental Fig. S5A; Yang et al. 2015). We found that SG RBP CLIP sites showed a qualitative correlation with the enrichment of individual NORAD segments within SGs (Supplemental Fig. S5B), and that when we summed the total number of SG RBPs for each segment of NORAD there was a good correlation between SG CLIP sites and NORAD segment enrichment (Supplemental Fig. S5C). Taken together, these results are consistent with a model in which RBPs can act in tandem to define the RNA composition of SGs.

### Loss of G3BP1 and 2 does not globally affect RNA localization to SGs

**FIGURE 5.**
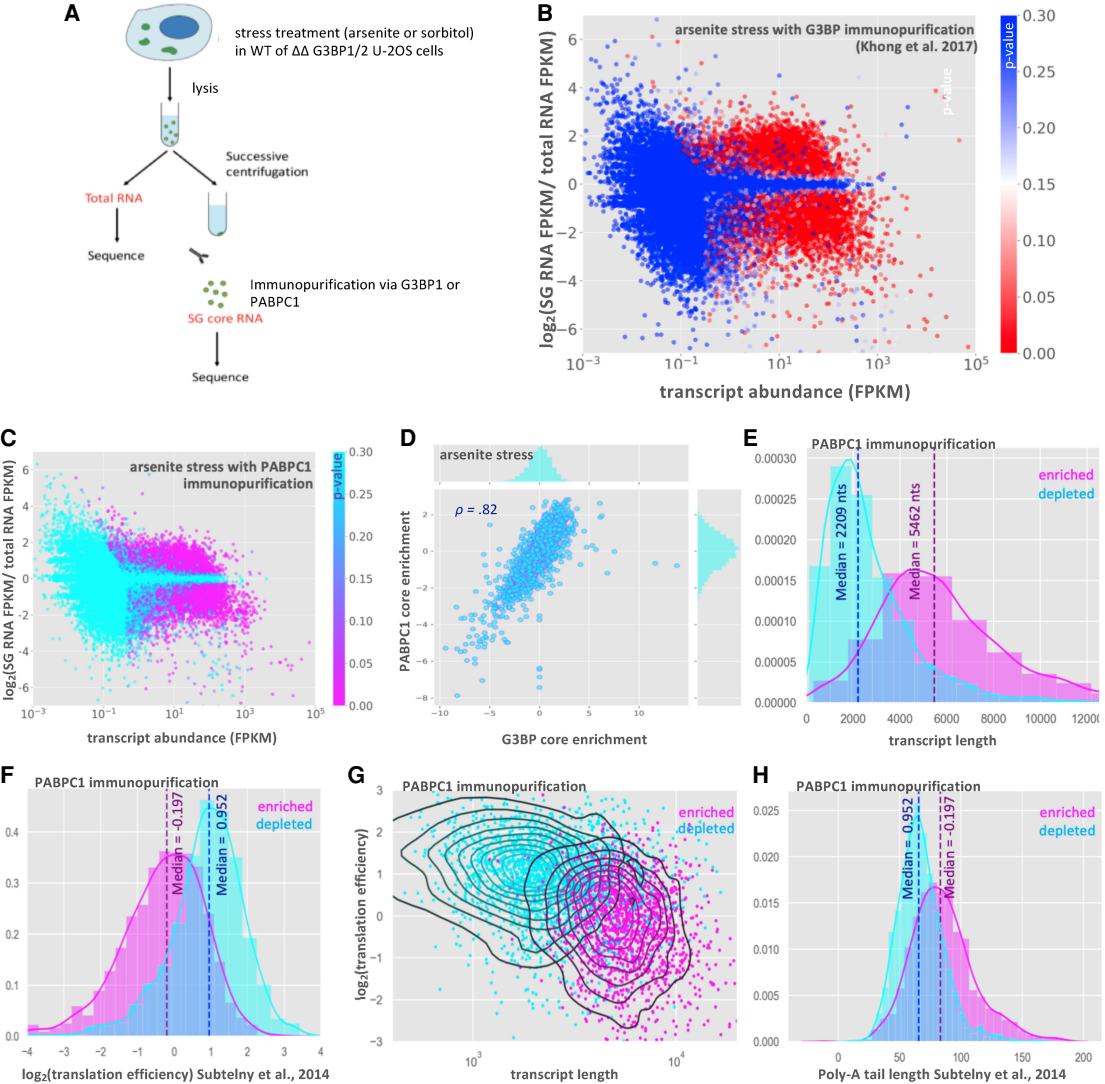
RNA-seq of the PABPC1 stress granule core transcriptome. (*A*) Schematic of SG core purification strategy in WT or ΔΔG3BP1/2 cells treated with either arsenite or sorbitol stress and using G3BP1 or PABPC1 for immunopurificaiton. (*B*) MA-plot showing stress granule enrichment versus abundance from previous work examining the SG transcriptome via G3BP1 immunopurification during arsenite stress (Khong et al. 2017). (*C*) MA-plot showing stress granule enrichment versus abundance from PABPC1 immunopurification during arsenite stress. (*D*) Scatterplot of RNA enrichment in PABPC1 cores versus enrichment in G3BP cores during arsenite stress. (*E*) Histogram showing distribution of SG-enriched and SG-depleted transcripts with respect to transcript length (purified with PABPC1). (*F*) Same as *E* but for translation efficiency. (*G*) Scatterplot of translation efficiency versus transcript length with kernel density estimate overlay. Color-coded by enrichment/depletion in SGs. (*H*) Same as *E*, but for poly(A) tail length.

Our modeling of SG-mRNA interactions suggests that RBPs act in tandem to contribute to RNP enrichment within SGs. If this model is true, we would anticipate that deletion of individual SG RBPs, such as G3BP, should have a limited effect on the recruitment of transcripts. To test this prediction, we desired to purify and determine the SG transcriptome from cells with and without G3BP1/G3BP2. In order to perform this experiment, we needed to purify SGs using a different SG component than G3BP1, which was used in earlier work (Khong et al. 2017), and under sorbitol stress condition, where G3BP1 and G3BP2 are not required for SG formation (Kedersha et al. 2016). Thus, we first tested whether immunopurification using an antibody to the SG component PABPC1 yielded a similar SG transcriptome as that seen with GFP-G3BP1 under arsenite stress (Fig. 5A–D; Khong et al. 2017).

Comparison of the RNAs enriched in SG cores showed that enrichment scores from PABPC1-purified cores and GFP-G3BP1-purified cores had a strong linear correlation (Fig. 5D, Pearson's *r* = 0.82). Total RNA and SG core purification via PABPC1 pulldown yielded reproducible transcriptomes (Supplemental Fig. S6). SG RNA transcriptomes based on the purification of SG cores with PABPC1 antibody showed little similarity to total RNA transcriptomes, with 3251 transcripts enriched in SGs (*P* < 0.01), and 3693 transcripts depleted from SGs (*P* < 0.01) (Fig. 5C). Thus, PABPC1 pulldown identifies a population of RNAs that strongly overlaps with the SG transcriptome identified by GFP-G3BP1 pulldown. Consistent with PABPC1 and GFP-G3BP1 purification identifying similar RNAs, we observed that in both cases, RNAs enriched in PABPC1-containing SGs are biased toward long, poorly translated RNAs (Fig. 5E–G; Subtelny et al. 2014; Khong et al. 2017). Additionally, we observe that enriched RNAs tend to have longer poly(A) tails (data obtained from Subtelny et al. 2014), which could be explained by transcripts with longer poly(A) tails having less efficient translation rates, lower overall abundance, or by the increased length of these transcripts (Fig. 5H; Supplemental Fig. S7; Lima et al. 2017). Taken together our findings suggest that PABPC1 and G3BP1 SG cores share a similar RNA composition and that the RNAs that copurify with PABPC1 and G3BP1 cores have similar physical properties.

**FIGURE 6.**
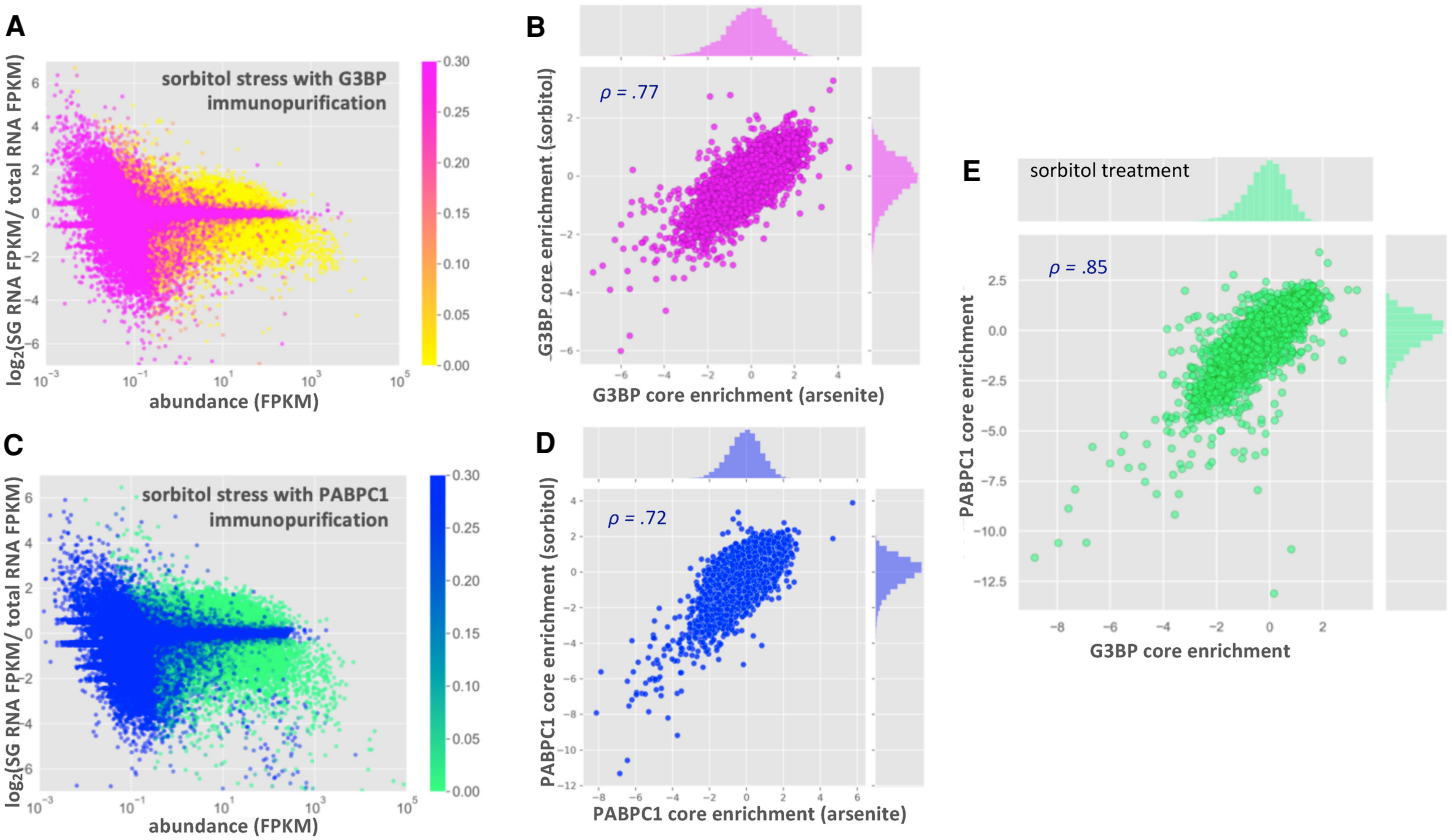
RNA localization to stress granule cores is conserved between sorbitol and arsenite stress. (*A*) MA-plot showing stress granule enrichment versus abundance for G3BP1 purified SGs during sorbitol treatment (color-coded by p-value). (*B*) Scatterplot showing correlation between G3BP core enrichment scores in sorbitol versus arsenite stress. (*C*) MA-plot showing stress granule enrichment versus abundance for PABPC1 purified SGs during sorbitol treatment (color-coded by p-value). (*D*) Scatterplot showing correlation between RNA enrichment scores from PABPC1 core purification in sorbitol versus arsenite stress. (*E*) Scatterplot showing correlation between enrichment scores from PABPC1- and G3BP- purified SG cores during sorbitol stress.

Since ΔΔG3BP1/2 cell lines form SGs during sorbitol treatment, we planned on analyzing SG transcriptomes from sorbitol-treated cells with and without G3BP1/2 expression. Before sequencing PABP-containing SGs from WT and ΔΔG3BP1/2 cells during sorbitol stress, we first examined whether the RNA composition of sorbitol-induced SGs was similar to arsenite-induced SGs. Thus, we compared the transcriptome of SG cores purified via PABPC1 and GFP-G3BP1 immunopurification during hyperosmotic stress induced by sorbitol (Fig. 6; Supplemental Figs. S8,S9; Dewey et al. 2011; Kedersha et al. 2016).

Purification of GFP-G3BP1 SGs under sorbitol stress yielded a very similar transcriptome to that observed under arsenite stress, with 2829 significantly enriched and 3721 significantly depleted transcripts (Fig. 6A,B). This is in agreement with the previous observations that the SG transcriptome is conserved between multiple stresses (Khong et al. 2017; Namkoong et al. 2018). We also observed that the SG transcriptome based on PABPC1 immunopurification is largely conserved between arsenite and sorbitol stresses and is similar to the SG transcriptomes defined by G3BP1 immunopurification (Fig. 6C–E). Taken together, our results indicate that the SG transcriptome is highly similar between arsenite and sorbitol stress conditions, and that the mRNAs pulled down are independent of the SG protein used for the affinity purification.

**FIGURE 7.**
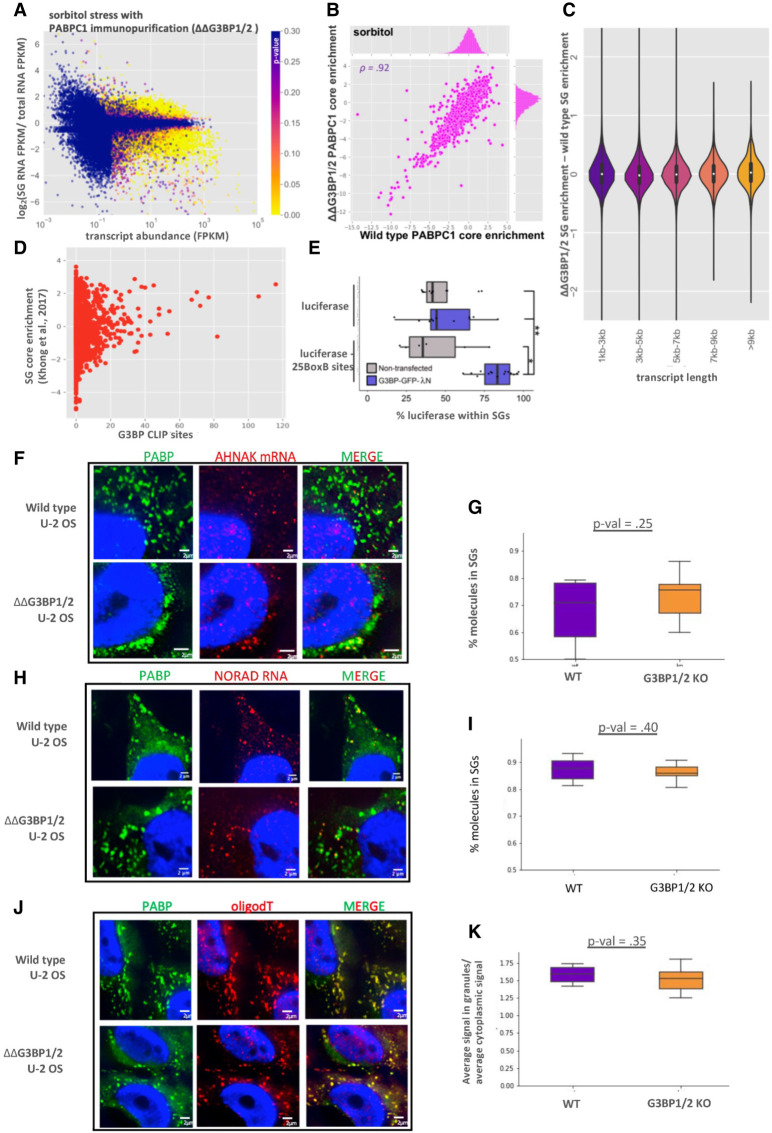
Global RNA localization to stress granule cores is independent of G3BP. (*A*) MA-plot showing stress granule enrichment versus abundance for PABPC1 purified SGs during sorbitol treatment in ΔΔG3BP1/2 cells. (*B*) Scatterplot depicting the enrichment of transcripts in PABPC1 stress granule cores in ΔΔG3BP1/2 versus WT U-2 OS cells during sorbitol stress. (*C*) Violin plots depicting ΔΔG3BP1/2—WT stress granule enrichment scores for all transcripts, binned by length. (*D*) Scatterplot depicting the fraction of a transcript that is localized to SGs during arsenite stress versus the number of G3BP eCLIP peaks for that transcript. (*E*) Boxplot showing the localization to SGs of luciferase reporter RNAs with 0 or 25 BoxB sites during sorbitol stress in nontransfected and G3BP-GFP-λN transfected cells. (*) *P* < 0.01, (**) *P* < 0.001. (*F*) smFISH of AHNAK mRNA during sorbitol stress in WT and ΔΔG3BP1/2 cells. (*G*) Boxplot quantifying the fraction of AHNAK cytoplasmic transcripts in SGs in WT and ΔΔG3BP1/2 cells. (*H*) smFISH of NORAD mRNA during sorbitol stress in WT and ΔΔG3BP1/2 cells. (*I*) Boxplot quantifying the fraction of NORAD in SGs in WT and ΔΔG3BP1/2 cells. (*J*) FISH of poly(A) RNA during sorbitol stress in WT and ΔΔG3BP1/2 cells. (*K*) Boxplot quantifying average intensity of poly(A) signal inside SGs versus average intensity of poly(A) signal in the cytoplasm.

We then examined whether G3BP1 and G3BP2 affected the mRNAs partitioning into SGs during sorbitol stress. To test this possibility, we purified sorbitol-induced SG cores from WT and ΔΔG3BP1/2 U-2 OS cells using antibodies to PABPC1 (Supplemental Figs. S9,S10). Strikingly, sorbitol-induced SGs contained a similar transcriptome regardless of whether cells expressed or lacked G3BP1 and G3BP2 (Fig. 7A,B). The enrichment scores for these transcriptomes showed a strong linear correlation (*R* = 0.94), suggesting that the enrichment of RNA in SGs is largely independent of G3BP during sorbitol stress. We interpret this observation to argue that G3BP1 and G3BP2 do not generally affect the mRNAs recruited to SGs.

Interactions between G3BP and mRNA might act in concert with other RBP–RNA, or RNA–RNA interactions to drive RNAs into SGs (Khong et al. 2017; Van Treeck et al. 2018). In this view, G3BP would only affect the recruitment of mRNAs to SGs that were on the cusp of SG enrichment. In order to examine this possibility, we calculated the change in ΔΔG3BP1/2 SG enrichment scores versus WT enrichment scores as a function of RNA length. If G3BP acted in concert with other RNA–RNA and RNA–RBP interactions to drive localization to SGs, one would anticipate that G3BP would only have an observable effect for transcripts of shorter lengths, which have fewer interactions. We observed similar SG enrichment between WT and ΔΔG3BP1/2 cell lines for RNAs of any length (Fig. 7C). Thus, we observed no significant impact of G3BP1 and G3BP2 on mRNA targeting to SGs even when binning for different lengths of mRNAs.

Another possibility is that only transcripts that exhibit G3BP binding capability would show differential recruitment to SGs. Thus, we utilized data from a recent eCLIP study which examined G3BP eCLIP targets (Van Nostrand et al. 2020). However, we observed that G3BP binding as assessed by eCLIP and SG enrichment were only weakly positively correlated (Fig. 7D). Moreover, G3BP target transcripts showed no altered localization in ΔΔG3BP1/2 cells, nor when we limited the analysis to short transcripts (Supplemental Fig. S11).

G3BP is required for SG formation during arsenite stress, but not sorbitol stress. Thus, one possibility is that G3BP simply does not have any influence over the partitioning of RNAs during sorbitol stress. To test this possibility, we examined if tethered G3BP1 could target the luciferase mRNA to SGs under sorbitol stress in a manner similar to what was observed under arsenite stress. We observed that G3BP1 tethering led to a significant and reproducible increase in luciferase localization to SGs during sorbitol stress (Fig. 7E). Thus, G3BP1 can still artificially drive SG enrichment of a reporter mRNA even in sorbitol stress wherein G3BP is not required for SG formation.

A final possibility for why the SG transcriptome does not change in the ΔΔG3BP1/2 cell lines is that depletion of G3BP affects all transcripts equally, which might be missed in our sequencing data. Thus, we examined whether global RNA recruitment to SGs was altered in ΔΔG3BP1/2 cells. By smFISH, we detected no discernable difference in the localization of AHNAK (Fig. 7F,G). This is in spite of the fact that AHNAK has over 70 predicted G3BP CLIP sites (Fig. 7D). This suggests that even transcripts with many G3BP sites may see modest changes in partitioning, likely due to compensating interactions that extremely large mRNAs like AHNAK (18 kb) are capable of forming. Both NORAD and total poly(A)+ signal showed a slight reduction in SG partitioning in ΔΔG3BP1/2 cells (Fig. 7H–K), although these reductions did not achieve statistical significance. Both AHNAK and NORAD transcripts lie in the plateau region of our mathematical model of SG interactions (Fig. 4). Thus, as we predicted from our previous mathematical modeling of SG enrichment, SG-enriched transcripts containing large numbers of interactions with SG-enriched RBPs should be insensitive to deletion of any single RBP. These findings argue that G3BP does not strongly affect the SG transcriptome. More broadly, this suggests that individual interactions between an mRNP and a SG that are of a similar strength as G3BP interactions with SGs will have minimal effects on mRNP partitioning into SGs. Therefore, RNA localization to SGs largely arises through the synergistic effects of multiple interactions acting in concert.

## DISCUSSION

In this work, we present evidence that specific RNA-binding proteins can localize mRNPs into SGs. The critical observation is that tethered G3BP1, TIA1, or FMRP can increase the partitioning of the luciferase mRNA into SGs (Figs. 2, 3). Although it has been widely anticipated that RBPs can target mRNAs to SGs, to our knowledge this observation provides the first demonstration of this principle. We cannot rule out the possibility that the increased localization of reporter RNAs within SGs may also be due to seeding of SGs at the site of the reporter RNA due to the creation of a high local concentration of a SG component. We anticipate that both seeding and active recruitment contribute to enhanced localization of our reporter RNAs in SGs. Possible mechanisms by which mRNAs could be targeted to SGs by G3BP1 or TIA1 specifically include the formation of G3BP1 dimers (Kedersha et al. 2016), or interactions between the TIA1 prion-like domain (Gilks et al. 2004).

We present two lines of evidence that the partitioning of an mRNP into SGs will be based on multiple elements acting in an additive manner. First, we observed that multiple elements within the NORAD RNA were sufficient to increase the SG enrichment of a luciferase reporter RNA (Fig. 1). Second, we demonstrated that the ability of tethered G3BP1 to target the reporter mRNA to SGs was dose-dependent (Fig. 2). The ability of protein interactions to target mRNPs to SGs suggests that the partitioning of an mRNP into a SG will be a summation of protein–protein, protein–RNA, and RNA–RNA interactions between an individual mRNP and the SG. This is directly analogous to the hypothesis that SGs form through various combinations of interactions between different mRNPs (Van Treeck et al. 2018). Thus, the recruitment of mRNPs into SGs reflects the summation of a number of interactions between individual mRNPs.

The recruitment of RNPs can be considered a simple equilibrium binding reaction with the partitioning of the RNP being proportional to e^−ΔG/RT^. Moreover, we observed that the SG enrichment of individual RNPs was correlated with the number of interactions in a logarithmic manner (Fig. 4). This is consistent with the model where the energetics of each individual interaction sum together to give an overall ΔG for SG partitioning as predicted by simple equilibrium binding.

By using some reasonable assumptions, we are able to make estimations of the number of interactions a given RNP will have with SGs and its partition coefficient. In this analysis, we estimate the number of interactions based on their similarity to a G3BP1 interaction. While this is undoubtedly an over-simplification, it allows for the first estimation of how many interactions RNPs have within SGs. Moreover, we note that tethered G3BP1 and TIA1 gave similar degrees of SG partitioning of the luciferase mRNA with 25 BoxB sites, suggesting that at least these two proteins have similar interactions with SGs. These estimates suggest that mRNAs with <20% of their mRNAs in SGs have one to five predicted RNP–SG interactions, while mRNAs that partition greater than 50% of their mRNAs in SGs have over 15 predicted RNP–SG interactions. Although these are crude estimates these numbers make two important points. First, the number of interactions a highly enriched RNP has in SGs is quite high. For example, we estimate that AHNAK mRNA will have 97 predicted RNP–SG interactions. This highlights the second important conclusion: Any given interaction only makes a small contribution to the overall enrichment of mRNAs in SGs. For example, for the AHNAK mRNA we estimate removing a single interaction between the mRNP and SG would alter the enrichment by 0.2%.

As a test of this rationale, we examined the global RNA composition of SGs in cells lacking the highly abundant SG RBPs G3BP1 and 2. In agreement with our hypothesis, we found that WT and ΔΔG3BP1/2 cell lines contain a similar SG transcriptome (Fig. 7). This observation was confirmed by smFISH for AHNAK, NORAD, and by oligodT staining.

Taken together, we propose a model in which multiple RBP–RNA and RNA–RNA interactions act synergistically to define the RNA composition of SGs (Supplemental Fig. S12). In this model, deletion or depletion of any single RBP would have a negligible effect on RNA localization to SGs, which is supported by the evidence that G3BP1 and G3BP2 deletions had essentially no effect on the SG transcriptome (Fig. 7). Similarly, cells lacking Pumilio proteins can still accumulate NORAD in SGs, based on the sequencing of a heavy RNP containing fraction (Namkoong et al. 2018). This is likely due to the ability of other RBPs and RNA–RNA interactions to compensate for the absence of G3BP or Pumilio. It remains a challenging and technical feat to distinguish between the role of each type of interaction and SG partitioning. We presume that some RNPs will be driven to SGs primarily through RNA–RNA interactions while others may depend more heavily on the multivalent interactions of their constituent proteins. The fact that the RNA composition of SGs is largely conserved between different stresses (Fig. 6; Khong et al. 2017; Namkoong et al. 2018) also supports our estimations. Since some proteins are only recruited to SGs under specific stresses (Buchan and Parker 2009; Markmiller et al. 2018), the loss of these potential interactions in SGs has little effect on the global RNA composition of SGs. Thus, there are likely multiple synergistic/redundant interactions that lead to RNA enrichment in SGs. This is not to say that individual RBPs cannot modulate the ability of an RNA to enrich in SGs. Indeed, increasing the number of RBPs on a transcript can lead to the enhanced enrichment of that transcript in SGs (Figs. 2, 3). We hypothesize that other SG RBPs should also be able to modulate the recruitment of transcripts similarly to G3BP tethering. It remains possible that there will be some molecular interactions between RNPs and SGs that are much stronger than the average G3BP1-SG or TIA1-SG interaction we define here and will therefore have a larger effect on the SG partitioning of an individual mRNP.

Three recent studies focused on the importance of G3BP in the formation of stress granules (Guillén-Boizet et al. 2020; Sanders et al. 2020; Yang et al. 2020). All three commented on the ability of G3BP to modulate valency either through RNA-induced conformational switching of the protein through intramolecular interactions featuring the IDR and RBD (Guillén-Boizet et al. 2020; Yang et al. 2020) or through valence capping via the interaction of G3BP with other less-valent proteins (Sanders et al. 2020). An apparent conundrum from these observations is that G3BP1 is required for SG formation in many stresses, but its absence does not notably alter the transcriptome of SGs. We suggest that the resolution to this conundrum is due to the fundamental differences in the kinetics of SG assembly as compared to the recruitment of individual mRNPs into SGs. The formation of SGs is a cooperative process wherein the average interaction between mRNPs will set a critical threshold above which mRNP nucleation into SGs will initiate, followed by recruitment of additional mRNPs to form SG cores. If the average interaction between mRNPs is altered even by 10% this can shift the system into a region of nonassembly due to the highly cooperative nature of SG cores, which contain 20–70 mRNAs (Khong et al. 2017). Thus, SG assembly is highly cooperative and very sensitive to the average interaction strength between individual mRNPs. Such highly cooperative assembly, and sensitivity to small changes in average interactions is a general property of any large assembly made up of multiple components, which provides numerous opportunities for the regulation of higher scale assembly. In contrast, the recruitment of an individual mRNP into an existing SG can be considered a simple equilibrium binding reaction without a cooperative effect of changes in mRNP affinities such that changes in K_D_s have modest effects on mRNA partitioning.

This analysis makes several predictions. First, components required for SG formation can halt overall assembly without substantially affecting the transcriptome of SGs in cases where they do form. Second, increasing SG formation artificially (e.g., by inhibiting eIF4A [Tauber et al. 2020]; or by overexpressing TIA1) will still generally lead to the same mRNAs accumulating in SGs. Third, genetic or pharmacological manipulations that alter the initiation events, or average interactions between mRNPs, even by a small amount, can prevent SG formation.

## MATERIALS AND METHODS

### Plasmid construction

For a list of plasmids constructed for use in this manuscript, as well as oligos used for plasmid construction, please see Supplemental Table S3. Tet-inducible Luciferase reporter was a gift from Moritoshi Sato (Addgene plasmid # 64127; http://n2t.net/addgene:64127; RRID:Addgene_64127, Nihongaki et al. 2015). BoxB repeats were cloned out of pCMV5-25BoxB, which was a gift from Maria Carmo-Fonseca (Addgene plasmid # 60817; http://n2t.net/addgene:60817; RRID:Addgene_60817, Martin et al. 2013) and placed into the 3′UTR of the luciferase reporter. The vector used as the backbone for AAVS targeting of Cas9 was pRP2855, an AAVS-TDP43 plasmid, in which the TDP43 was excised by restriction digest and replaced with luciferase constructs. G3BP-GFP-λN and GFP-λN plasmids were gifts from Richard Lloyd's laboratory. FMRP was cloned out of pFRT-TODestFLAGHAhFMRPiso1, a gift from Thomas Tuschl (Addgene plasmid # 48690; http://n2t.net/addgene:48690: RRID:Addgene_48690, Ascano et al. 2012). NORAD-PREmut plasmid was a gift from Josh Mendel's laboratory and placed into the 3′-UTR of the luciferase reporter.

### Genomic integration into AAVS locus

Cells were transfected with 1 µg CRISPR/Cas9 plasmid (pRP2854) in conjunction with 1 µg appropriate luciferase reporter construct (pRP2856, pRP2873, and pRP2874) using Jet Prime reagent. Transfection of pRP2854 alone was used as a negative control. Twenty-four hours following transfection, cells were split from a six-well plate to a 10 cm dish. After another 24 h, media was replaced with new media containing 1 µg/mL puromycin to begin selection for cells with genomic integration. Following 24 h with puromycin selection, media was replaced with fresh puromycin. After all cells were dead in the negative control plate, media was replaced with fresh media lacking puromycin for 48 h. This is an optional step to help get rid of any residual plasmid. Puromycin was then added for another 48 h to finalize the selection. Single colony selection was not done for these experiments.

### Stellaris smFISH probes

Custom Stellaris smFISH probes against AHNAK, NORAD, and firefly luciferase transcripts were designed with Stellaris RNA FISH Probe Designer (Biosearch Technologies), available online at http://www.biosearchtech.com/stellaris-designer (version 4.2). AHNAK and NORAD smFISH probes, labeled with Quasar 670 dye, and firefly luciferase probes, labeled with Quasar 570, were ordered from Stellaris (Biosearch Technologies).

### Sequential IF and FISH

Sequential immunofluorescence and smFISH on fixed U-2 OS cells was performed with Stellaris buffers or homemade buffers (Dunagin et al. 2015) according to the manufacturer's protocol: (https://biosearchassets.blob.core.windows.net/assets/bti_custom_stellaris_immunofluorescence_seq_protocol.pdf).

Briefly, U-2 OS cells were seeded on sterilized coverslips in six-well tissue culture plates. At ∼80% confluency, media was exchanged 1 h before experimentation with fresh media. After stressing cells (see section “Stress conditions”), the media was aspirated and the cells were washed with prewarmed 1× PBS. The cells were fixed with 500 µL 4% paraformaldehyde for 10 min at room temperature. After fixation, cells were washed twice with 1× PBS, permeabilized in 0.1% Triton X-100 in 1× PBS for 5 min and washed once with 1× PBS.

For IF detection, coverslips were incubated in primary antibody for 1 h. Coverslips were washed three times with 1× PBS for 10 min each wash. Then cells were incubated in secondary antibody (Thermo Fisher Scientific A-31553). Again, coverslips were washed three times with 1× PBS for 10 min each wash. Then, cells were treated with smFISH Buffer A for 5 min. Coverslips were transferred to a humidifying chamber with smFISH probes and placed in the dark at 37°C for 16 h. Coverslips were placed in Buffer A for 30 min in the dark, washed with Buffer B for 5 min and placed onto a slide with VECTASHIELD Antifade Mounting Medium with DAPI (Vector Labs, H-1200). For assays requiring quantification of smFISH probes in stress granules, VECTASHIELD Antifade Mounting Medium without DAPI was used (Vector Labs, H-1000).

In order to maintain consistency, the same protocol was utilized in IF only experiments; however, the portions of the protocol calling for smFISH were omitted. Antibodies that were used include PABP (Abcam ab21060), G3BP (Abcam ab56574). In all imaging experiments at least 10 cells were imaged.

smFISH probes were labeled based on a recent protocol (Gaspar et al. 2017). Oligonucleotides used for smFISH were designed using the Stellaris Probe Designer (https://www.biosearchtech.com/support/education/stellaris-rna-fish). Briefly, oligos were labeled by incubating 4 µL of 200 µM pooled oligonucleotides (Integrated DNA Technologies), 1 µL of TdT (Thermo Fischer Scientific EP0161), and 6 µL of ddUTP (Axxora JBS-NU-1619-633) fluorophore for 8 h. After 8 h, another 1 µL of TdT enzyme was added and the reaction was allowed to continue overnight. Probes were then ethanol precipitated by adding 164.5 µL of nuclease free water, 0.5 µL of 0.5 mg/mL linear acrylamide, 20 µL of 3 M sodium acetate (pH 5.5) and 800 µL of prechilled 100% ethanol. This mixture was then placed at −80°C for at least 20 min. Labeled oligos were pelleted by centrifugation at 16,000*g* at 4°C. Probes were then washed three times by adding 1 mL of 80% ethanol and centrifugation at 16,000*g* at 4°C. After washing, the pellet was allowed to air dry for 10 min and probes were resuspended in 25 µL of nuclease free water.

### Microscopy

Fixed U-2 OS cells stained by immunofluorescence and smFISH, were imaged using a wide field DeltaVision Elite microscope (Applied Biosystems) with a 100× objective and a PCO Edge sCMOS camera. At least five images with 20 *Z*-sections were taken for each experiment. All images in the manuscript are processed by FIJI (Schindelin et al. 2012) or Imaris (Bitplane).

### Imaris identification of smFISH spots

To measure the fraction of smFISH spots in stress granules, deconvolved images were analyzed using Bitplane Imaris image analysis software as described in detail in a previous manuscript (Khong et al. 2018). In short, images were first renamed so that the tallying of cells was blind. This accounts for the variability in the number of cells counted for each condition. We did ensure that at least 10 cells were counted for each condition. Cells were manually segmented to ensure counting of transfected and nontransfected cells. Cells that were saturated in the GFP channel under our imaging conditions were considered to be overexpressing G3BP, for example, and were excluded from analysis. Imaris can be used to automatically segment SGs as well as identify smFISH spots from each cell taking into account the Z-plane.

### U-2 OS growth conditions and reagents

Human osteosarcoma U-2 OS cells expressing G3BP1-GFP (Paul Taylor Laboratory), U-2 OS cells, and U-2 OS ΔΔG3BP1/2 (Kedersha et al. 2016) were used in all experiments. All cells were maintained in DMEM with high glucose, 10% fetal bovine serum, and 1% penicillin/streptomycin at 37°C/5% CO_2_.

### Isolation of RNA from U-2 OS cells and SG cores for RNA-sequencing

Parental and ΔΔG3BP1/2 U-2 OS cells expressing G3BP1-GFP were grown to 85% confluency in three 500 cm^2^ TC-treated culture dishes (Thermo Fisher Scientific, 07-200-599). One hour prior to stress, cell culture media was exchanged with fresh media. Cells were then stressed with either NaAsO_2_ or sorbitol (see section “Stress conditions”). After stress, cells were washed once with media, transferred to falcon tubes, and pelleted at 1500*g* for 3 min. Upon aspirating the media, the pellets were immediately flash-frozen in liquid N_2_ and stored at −80°C until isolation of mammalian SG cores was performed. PABPC1 SG cores were purified as previously described for the purification of G3BP cores; however, instead of using anti-GFP for the pulldown of SG cores, 20 µL of anti-PABPC1 (ab21060) was used (Khong et al. 2017, 2018). For all experiments, biological triplicates were acquired for both SG-purified RNA and total RNA except in the case of PABPC1 SG RNA purified during sorbitol stress, in which duplicates were acquired (one of the replicates was excluded because it showed poor quality control metrics and, therefore, showed little similarity to the other biological replicates).

### Stress conditions

To examine mRNA localization during stress, we used the following stress conditions. For arsenite stress experiments, cells were treated with 0.5 mM sodium arsenite (Sigma-Aldrich S7400) for 1 h. For osmotic stress, cells were stressed in 0.5M D-sorbitol (Sigma-Aldrich S1876) for 2.5 h. Cells were fixed after the completion of each stress with 4% paraformaldehyde (Fisher Scientific NC0179595).

### Library construction and RNA-sequencing

RNA quality was assessed by TapeStation analysis at the Biofrontiers Institute Sequencing Facility. Paired-end cDNA libraries were prepared at the Biofrontiers Institute Sequencing Facility using the KAPA HyperPrep with RiboErase. cDNA libraries were sequenced on a NextSeq High output 150 cycle (2 × 75).

### Sequencing data analysis

Read quality was assessed using fastqc. An index genome was acquired from GENCODE (Release 19 GRCh37.p13). Reads were aligned using hisat2. Differential expression analysis was performed using Cuffdiff (version 2.2.1) with the default parameters (Trapnell et al. 2013). Gene Ontology analysis was performed using the gene ontology consortium (http://www.geneontology.org/). Transcript lengths were acquired from Ensembl's Biomart Tool. All sequencing data can be found at NCBI GEO GSE119977.

### Mathematical modeling

Curve fitting was performed using Python's scikit-learn package. The median percent enrichment within stress granules was plotted against the number of box-b sites. The initial number of potential interactions the luciferase can form with SGs, which we refer to as *n*, was allowed to vary from one to 10 and the best fit was chosen by examining the Pearson's *r* value of each line. The data was linearized by performing a log-transformation of the data and *r* values, slope, and intercept positions were calculated using the sklearn package in Python. The code for this analysis is available at the following github repository: https://github.com/tmatheny/stress_granule.

## SUPPLEMENTAL MATERIAL

Supplemental material is available for this article.
